# Stent deformation after surgical aortic valve replacement in a patient undergoing percutaneous coronary intervention of proximal right coronary artery

**DOI:** 10.1093/ehjcr/ytad633

**Published:** 2023-12-19

**Authors:** Takumi Osawa, Hidetaka Nishina

**Affiliations:** Department of Cardiology, Tsukuba Medical Center Hospital, 1-3-1 Amakubo, 3058558 Tsukuba, Japan; Department of Cardiology, Tsukuba Medical Center Hospital, 1-3-1 Amakubo, 3058558 Tsukuba, Japan

**Figure:**
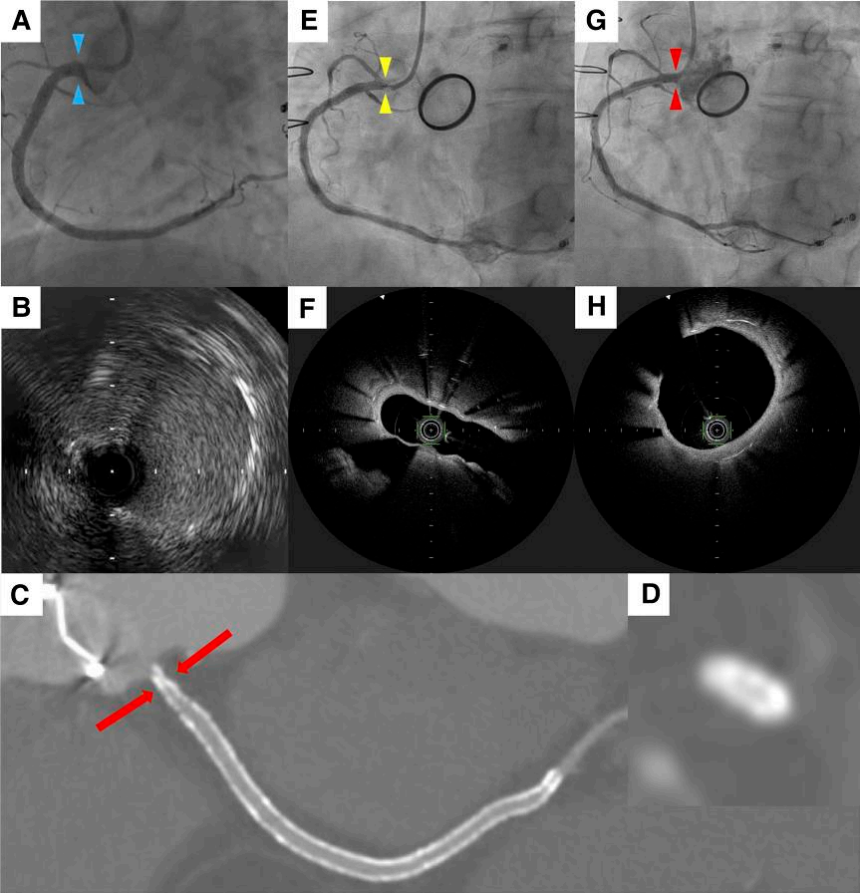


A 71-year-old woman with effort angina was admitted to our institution. She was previously diagnosed with hypertension and hyperlipidaemia. Eight years prior to admission, she underwent percutaneous coronary intervention (PCI) of the right coronary artery (RCA) ostium with a drug-eluting stent (Promus Premier 3.5/38 mm, Boston Scientific, Natick, MA, USA) (*Panels A* and *B*). Three years ago, she developed heart failure due to severe aortic regurgitation and underwent surgical aortic valve replacement (SAVR; 18 mm ATS AP valve, ATS Medical Inc., Minneapolis, MN, USA) under cardiopulmonary bypass. Due to frequent effort angina, we performed the coronary computed tomography angiography, revealing luminal narrowing due to severe stent deformation at the proximal RCA (*Panels C* and *D*). Invasive coronary angiography showed angiographically equivocal stenosis of the RCA ostium (*Panel E* and Supplementary data online, *Video S1*). Optical frequency domain imaging (OFDI) (FastView, Terumo, Tokyo, Japan) verified stent deformation at the RCA ostium without intimal proliferation (*Panel F* and Supplementary data online, *Video S2*). The patient underwent PCI of the RCA ostium using paclitaxel-coated balloon (SeQuent Please, 4.0/20 mm, B. Braun Melsungen, Germany) (*Panel G*). The OFDI confirmed re-expansion of the deformed stent (*Panel H*).

The patient remains free of angina after the procedure. The possible mechanisms of stent deformation were (1) influence during knotting of the aortic valve prosthesis, (2) application of cardioplegia to the RCA ostium, and (3) effects of cardiopulmonary withdrawal. This case illustrates a rare cause of iatrogenic coronary ostial stenosis due to stent deformation after cardiac surgery.

## Supplementary Material

ytad633_Supplementary_Data

## Data Availability

Data sharing is not applicable to this article as no datasets were generated or analysed during the current case report.

## Supplementary material

**Consent**: Informed consent has been obtained by the subject for the publication. The authors confirm the written consent for the submission and publication of this case report.

**Funding:** None.

## Panels

- Coronary angiography of the RCA after stenting of the proximal RCA
- Intravascular ultrasound after stenting of the RCA ostium
- Coronary computed tomography angiography of the RCA
- Short axis cross-section of coronary computed tomography of RCA
- Coronary angiography of the RCA after surgical aortic valve replacement
- OFDI of RCA showing stent deformation after surgical aortic valve replacement
- Coronary angiography of the RCA after PCI of the proximal RCA using the paclitaxel-coated balloon
- OFDI of the RCA showing dilation of stent struts following PCI of the proximal RCA.

